# Supplementary material for: Using machine learning to analyze mental health in distance education during the COVID-19 pandemic: an opinion study from university students in Mexico
Source: PeerJ Comput Sci. 2024 Aug 8;10:e2241. doi: 10.7717/peerj-cs.2241 (PMC11323079; doi:10.7717/peerj-cs.2241)
Supplement: Supplemental Information 10 [file peerj-cs-10-2241-s010.pdf]

Questionnaire

	Never	Sometimes	Always
Q1: Did I feel attention, commitment, and empathy from my teachers during the time they worked online?			
Q2: Did I feel too much workload in the activities in each of my subjects?			
Q3: Did the activities sent in your subjects overwhelmed, tired, or frustrated you emotionally or physically?			
Q4: Did you feel positive during your online learning? For examples, safe, satisfied, happy, relaxed and/or pleased			
Q5: Did you concentrate taking online classes?			
Q6: Did I feel stressed because of my time distribution when taking online classes?			
Q7: Did I feel that I was unfairly asked to continue or adapt my academic duties in the presence of the pandemic?			
Q8: Did you consider that your school performance during online classes accomplished what you were hoping for?			
Q9: Did you feel that COVID-19 psychologically affected your school performance?			

	Joy	Stress	Uncertainty
LQ: What would you feel if you were informed that classes would be online again?			
