# Supplementary material for: Using machine learning to analyze mental health in distance education during the COVID-19 pandemic: an opinion study from university students in Mexico
Source: PeerJ Comput Sci. 2024 Aug 8;10:e2241. doi: 10.7717/peerj-cs.2241 (PMC11323079; doi:10.7717/peerj-cs.2241)
Supplement: Supplemental Information 11 [file peerj-cs-10-2241-s011.pdf]

Cuestionario

	Nunca	Algunas veces	Siempre
Q1: ¿Sentí atención, compromiso y empatía por parte de mis docentes durante el tiempo que trabajaron en línea?			
Q2: ¿Sentí demasiada carga de trabajo en las actividades de cada una de mis materias?			
Q3: ¿Las actividades encargadas en tus materias te abrumaron, cansaron o frustraron emocional o físicamente?			
Q4: ¿Me sentí positivo durante mi aprendizaje en línea? Por ejemplo, seguro, satisfecho, feliz, relajado y/o complacido.			
Q5: ¿Te concentraste tomando clases en línea?			
Q6: ¿Me sentí estresado por la distribución de mi tiempo al tomar clases en línea?			
Q7: ¿Sentí que me pidieron injustamente que continuara o adaptara mis deberes académicos durante la pandemia?			
Q8: ¿Considero que mi desempeño escolar durante las clases en línea logró lo que esperaba?			
Q9: ¿Sentí que el COVID-19 afectó psicológicamente mi desempeño escolar?			

	Alegría	Estrés	Incertidumbre
LQ: ¿Qué sentirías si te informaran que las clases volverían a ser en línea?			
